# Reconstruction of the Doradinae (Siluriformes-Doradidae) ancestral diploid number and NOR pattern reveals new insights about the karyotypic diversification of the Neotropical thorny catfishes

**DOI:** 10.1590/1678-4685-GMB-2020-0068

**Published:** 2021-11-24

**Authors:** Fábio H. Takagui, Patrik Viana, Lucas Baumgärtner, Jamille A. Bitencourt, Vladimir Pavan Margarido, Roberto Laridondo Lui, Eliana Feldberg, Jose Luis Olivan Birindelli, Fernanda Simões Almeida, Lucia Giuliano-Caetano

**Affiliations:** 1 Universidade Estadual de Londrina, Departamento de Biologia Geral, Laboratório de Citogenética Animal, Londrina, PR, Brazil.; 2 Instituto Nacional de Pesquisas da Amazônia, Laboratório de Genética Animal, Manaus, AM, Brazil.; 3 Universidade Estadual do Oeste do Paraná, Centro de Ciências Biológicas e da Saúde, Laboratório de Citogenética, Cascavel, PR, Brazil.; 4 Universidade Estadual do Sudoeste da Bahia, Departamento de Ciências Biológicas, Laboratório de Citogenética, Jequié, BA, Brazil.; 5 Universidade Estadual de Londrina, Departamento de Biologia Animal e Vegetal, Museu de Zoologia, Londrina, PR, Brazil.; 6 Universidade Estadual de Londrina, Departamento de Biologia Geral, Laboratório de Genética e Ecologia Animal, Londrina, PR, Brazil.

**Keywords:** Karyotypic diversification, Cytotaxonomy, 5S rDNA, 18S rDNA, Heterochromatin

## Abstract

Doradinae (Siluriformes: Doradidae) is the most species-rich subfamily among thorny catfishes, encompassing over 77 valid species, found mainly in Amazon and Platina hydrographic basins. Here, we analyzed seven Doradinae species using combined methods (e.g., cytogenetic tools and Mesquite ancestral reconstruction software) in order to scrutinize the processes that mediated the karyotype diversification in this subfamily. Our ancestral reconstruction recovered that 2n=58 chromosomes and simple nucleolar organizer regions (NOR) are ancestral features only for Wertheimerinae and the most clades of Doradinae. Some exceptions were found in *Trachydoras paraguayensis* (2n=56), *Trachydoras steindachneri* (2n=60), *Ossancora punctata* (2n=66) and *Platydoras hancockii* whose karyotypes showed a multiple NOR system. The large thorny catfishes, such as *Pterodoras granulosus*, *Oxydoras niger* and *Centrodoras brachiatus* share several karyotype features, with subtle variations only regarding their heterochromatin distribution. On the other hand, a remarkable karyotypic variability has been reported in the fimbriate barbells thorny catfishes. These two contrasting karyoevolution trajectories emerged from a complex interaction between chromosome rearrangements (e.g., inversions and Robertsonian translocations) and mechanisms of heterochromatin dispersion. Moreover, we believe that biological features, such as microhabitats preferences, populational size, low vagility and migratory behavior played a key role during the origin and maintenance of chromosome diversity in Doradinae subfamily.

## Introduction

Cytogenetic studies have provided valuable information about the evolutionary trends and relationships in a range of vertebrate species, such as amphibians (Bruschi et al., 2019), reptiles (Viana et al., 2019, 2020), birds (Damas *et al*., 2019; Sigeman et al., 2019), mammals (Graphodastsky et al., 2011) and fish (Sember *et al*., 2018; Takagui et al., 2019). Different softwares for reconstruction of ancestral characters (e.g., Chromoevol, Mesquite) have been incorporated into cytogenetic analyses in recent years and provided a better understanding regarding the karyotype evolution in several organisms, as seen in plants (Burchardt *et al*., 2018), insects (Castillo et al., 2018; Micolino et al., 2019), birds (Damas *et al*., 2018) and mammals (Kim et al., 2017). 

Despite the paucity of studies involving this kind of evolutionary approach in fish, analysis combining cytogenetic data and reconstruction of ancestral features have emerged in recent years (Cardoso *et al*., 2018; Terra et al., 2019). Therefore, these studies demonstrate the efficiency of combined analysis between robust phylogenetic relationships and pre-establishes chromosomal patterns in generating accurate estimates of ancestral chromosomal states in fish, especially in groups that possess a huge karyotype diversity, as for instance the Doradidae family.

Within Neotropical Siluriformes, Doradidae stands out as one of the most diverse and representative families, with over 96 species (Fricke et al., 2020), commonly known as thorny or spiny catfishes. They are a remarkable group, easily recognized by the presence of a single rows of scutes with thorns along the lateral line. Thorny catfishes are widely distributed across the largest hydrographic basins in South America, although the highest diversity is found in the Amazon and La Plata basins (Ferraris, 2007; Birindelli, 2014). The relationships among Doradidae species were already investigated through morphological and molecular data and the monophyly of this family as well as its subfamilies are usually corroborated by both approaches (Arce et al., 2013; Birindelli, 2014).

Doradidae is classified into three subfamilies: Wertheimerinae (3 species), Astrodoradinae (15 species), and Doradinae (78 species) (Fricke et al., 2020). The latter, represents the most diverse of all subfamilies and includes large species that are found mainly in the main channel of large rivers and exhibit migratory behavior during reproduction, represented by species as *Pterodoras granulosus* Valenciennes, 1821, *Oxydoras niger* Valenciennes, 1821, *Centrodoras brachiatus* Cope, 1872, *Megalodoras uranoscopus* Eigenmann & Eigenmann, 1888, *Lithodoras dorsalis* Bleeker, 1862 (Goulding, 1980; Agostinho et al., 2003; Birindelli and Sousa 2017). On the other hand, Doradinae also includes tiny species, characterized by the presence of fimbriate barbels, such as *Hemidoras*, *Trachydoras*, *Ossancora* and *Tenellus* (Sabaj, 2005; Arce et al., 2013; Birindelli, 2014; Birindelli and Sousa 2017). The latter group, which has a wide morphological variability and behavioral lability, not only includes sedentary species but also others with high vagility (Sabaj, 2005; Birindelli and Sousa, 2017).

Karyotype data is available solely for 19 out of the 96 Doradidae species, most of them having 58 chromosomes, except for *Anadoras* sp. “araguaia” and *Trachydoras. paraguayensis* Eigenmann & Ward 1907 (2n=56 chromosomes), and *Ossancora punctata* Kner, 1853 (2n=66 chromosomes), the highest diploid number in the family to date. Additionally, a considerable cytogenetic variability is also observed in the structural level (i.e., karyotype formulas, heterochromatin patterns and rDNA sites distribution), supernumerary chromosomes, as seen in *Ossancora punctata*, *Pterodoras granulosus* and *Platydoras armatulus* Valenciennes, 1840 and a unique ZZ/ZW sex chromosome system in *Tenellus trimaculatus* Boulenger, 1898 (Table 1). Thus, it is believed that the origin of the current karyotype diversity in Doradidae has been assigned to numerical (Robertsonian translocations), structural (pericentric inversions) and different mechanisms of repetitive DNA dispersion (Baumgärtner et al., 2018; Takagui et al., 2019). 

To unravel the evolutionaty processes that drove the karyotype diversification of the Neotropical Doradidae and to better characterize its likely ancestral karyotype state, we applied an extensive suite of cytogenetic tools in a range of Doradinae subspecies, which allowed us to identify patterns of homologies and independent diversification in some particular clades of this subfamily. In addition, we also recovered ancestral features regarding the macro and micro karyotype structure based on a robust phylogeny, providing a better understanding about the karyotype evolution of the Neotropical thorny catfishes.

**Table 1 -:** Cytogenetic data available for the Neotropical freshwater fishes of Doradidae family.

GENERA/ SPECIES	2n	KARYOTYPE	Ag-NORs	18S rDNA	5S rDNA	REFERENCES
Wertheimerinae Subfamily						
*Wertheimeria maculata*	58	24m+14sm+8st+12a	Pair 20 (p arm)	-	-	Eler *et al*. (2007)
*Wertheimeria maculata*	58	24m + 12sm + 8st + 14st-a	-	Pair 22 (p arm)	Pair 22 (p arm)	Takagui *et al*. (2019)
*Kalyptodoras bahiensis*	58	24m + 12sm + 8st + 14st-a	-	Pair 22 (p arm)	Pair 22 (p arm)/ Pair 19 (p arm)	Takagui *et al*. (2019)
*Franciscodoras marmoratus*	58	24m + 12sm + 8st + 14st-a	-	Pair 22 (p arm)	Pair 22 (p arm)/ Pair 19 (p arm)	Takagui *et al*. (2019)
Astrodoradinae Subfamily						
*Anadoras* sp. “araguaia”	56	24m+10sm+8st+14a	Pair 28 (q arm)	Pair 28 (q arm)	Pair 15 (p arm)	Baumgärtner *et al*. (2018)
Doradinae Subfamily						
*Platydoras* cf. *costatus*	58	26m+16sm+4st+2a	Pair 20 (p arm)	-	-	Milhomem *et al*. (2008)
*Platydoras armatulus*	58	22m+14sm+18st+4a	-	-	-	Takagui *et al*. (2017a)
*Platydoras armatulus*	58	24m+14sm+20st	Pair 25 (p arm)	Pair 25 (p arm)	Pairs 18, 25	Baumgärtner *et al*. (2018)
*Pterodoras granulosus*	58	16m +16sm+14st+12a	-	-	-	Takagui *et al*. (2017a)
*Oxydoras niger*	58	20m+16sm+8st+14a	Pair 15 (p arm)	-	-	Fenocchio *et al*. (1993)
*Rhinodoras dorbignyi*	58	20m+20sm+4st+14a	Pair 16 (p arm)	-	-	Fenocchio *et al*. (1993)
*Rhinodoras dorbignyi*	58	18m+16sm+12st+12a	Pair sm (p arm)	-	-	Fenocchio *et al*. (1993)
*Rhinodoras dorbignyi*	58	24m+12sm+12st+10a	Pair 24 (p arm)	Pair 24 (p arm)	Pairs 18,24,26	Baumgärtner *et al*. (2018)
*Ossancora punctata*	66	12m+8sm+6st+40a	-	-	-	Takagui *et al*. (2017a)
*Ossancora eigenmanni*	58	30m+14sm+14st	Pair 17 (p arm)	Pair 17	Pairs 10, 17,23	Baumgärtner *et al*. (2018)
*Trachydoras paraguayensis*	56	32m+20sm+4st	sm Pair	-	-	Fenocchio *et al*. (1993)
*Trachydoras paraguayensis*	56	36m+16sm+4st	Pair 11 (Interstitial)	Pair 11 (Interstitial)	Pair 22	Baumgärtner *et al*. (2016)
*Tenellus leporhinus*	58	36m+18sm+4st	Pair 23 (q arm)	Pair 23	Pair 10	Takagui *et al*. (2017b)
*Tenellus trimaculatus*	58	♀ 21m+18sm+12st+7a ♂ 20m+18sm+12st+8a	Pair 22 (p arm)	Pair 22 (p arm)	Four sites	Takagui *et al*. (2017b)
*Tenellus ternetzi*	58	44m+12sm+2 a	Pair 24 (q arm)	-	-	Milhomem *et al*. (2008)
*Hassar orestis*	58	32m+20sm+6a	Pair 22 (p arm)	-	-	Milhomem *et al*. (2008)
*Hassar* cf*. orestis*	58	32m+18sm+8a	Pair 20 (p arm)	-	-	Milhomem *et al*. (2008)
*Hassar wilderi*	58	32m+16sm+10a	Pair 25 (p arm)	-	-	Eler *et al*. (2007)
*Hassar wilderi*	58	26m+20sm+12st	Pair 28 (p arm)	Pair 28 (p arm)	Four sites	Takagui *et al*. (2017b)
*Hassar* sp.	58	42m+14sm+2a	Pair 7 (p arm)	-	-	Milhomem *et al*. (2008)
*Leptodoras cataniae*	58	24m+16sm+14st+4a	Pair 23 (p arm)	Pair 23 (p arm)	Four sites	Takagui *et al*. (2017b)

Legend: 2n=diploid number; m=metacentric; sm=submetacentric; st=subtelocentric; a=acrocentric; Ag-NORs=Nitrate impregnation for detect the NORs sites; rDNA=ribossomal desoxiribonucleic acid; p arm=short arm; q arm=long arm. The information’s produced by dissertations, phD thesis or abstracts in national/international congresses were not included in the table.

## Material and Methods

### Species and collection sites

Our representative sampling encompassed a total of 35 individuals of seven different thorny catfish species from different Brazilian hydrographic basins. All specimens here analyzed were collected under permission granted by Instituto Chico Mendes de Conservação da Biodiversidade (ICMBio) number 11399-1. All procedures and experiments used in this study were approved, performed in accordance with all relevant guidelines and fulfill the rules of the Ethics Committee for Animal Use of the Londrina State University (Protocol: 60/2017). The individuals were properly identified by morphological criteria and subsequently deposited in the Museum of Zoology of the State University of Londrina (MZUEL), available online via SpeciesLink (Table 2).

**Table 2 -:** Information about the species under study, their sex, collection sites and Vouchers in Ichthyological Collections.

Species	Number of individuals	Localities	Coordinates	Vouchers
*Platydoras hancockii*	3 males	Negro River - Central Amazon basin	0°58’31.68’’S 62°55’40.79’’W	MZUEL17318
*Centrodoras brachiatus*	2 females	Solimões River - Central Amazon basin	3°14’28.32’’S 59°56’29.19’’W	MZUEL17831
*Pterodoras granulosus*	4 males / 2 females	Solimões River - Central Amazon basin	3°09’34.11’’S 59°54’04.34’’W	MZUEL 20294
*Ossancora punctata*	5 males/3 females	Miranda River - Middle Paraguay basin	19°31’25’’S 57°02’26”W	MZUEL12170
*Oxydoras niger*	6 females	Catalao Lake - Central Amazon basin	3˚09’49.8”S 59˚54’47.5”W	MZUEL17317
*Trachydoras steindachneri*	4 females / 1 male	Solimões River - Central Amazon basin	3°09’34.11’’S 59°54’04.34’’W	MZUEL17802
*Hemidoras stenopeltis*	3 females / 2 male	Negro River - Central Amazon basin	0°58’31.68’’S 62°55’40.79’’W	MZUEL17807

Legend: [S]= South; [W]= West; [MZUEL]= Museum of Zoology of Londrina State University; [MZUSP]= Museum of Zoology of Sao Paulo University.

###  Mitotic chromosomes preparations, chromosomal banding and Fluorescence *in situ* hybridization (FISH) 

All individuals were treated with an intraperitoneal injection of 2 mL (1 mL/50 g) body weight) of bacterial lysate Broncho-vaxom (7 mg/mL), to trigger an inflammatory response and hence increase the number of renal cells in mitotic division (Molina et al., 2010). The mitotic chromosomes were obtained from kidney cells according to Bertollo et al. (1978). Heterochromatin was detected according to Sumner (1972) with modification in the staining step (Giemsa was replaced by propidium iodide) according to Lui et al. (2012).

Fluorescence *in situ* hybridization (FISH) was performed according to Pinkel et al. (1986). The rDNA probes were obtained by Mini-Prep (i.e., extraction of plasmidial DNA), 18S rDNA probe from *Prochilodus argenteus* Spix & Agassiz, 1829 (Hatanaka and Galetti, 2004) and 5S rDNA from *Megaleporinus elongatus* Valenciennes, 1850 (Martins and Galetti, 1999). The rDNA probes were labelled by nick translation (Roche) (according to the manufacturer’s instructions) using biotin-16-dUTP or digoxigenina-11-dUTP. Hybridizations were conducted under a high stringency (77%). The detection of the signals was performed using anti-digoxigenin-rhodamine (Roche) and avidin-FITC (Sigma-Aldrich). The karyotype morphology analysis followed Levan et al. (1964), but modified as metacentric (m), submetacentric (sm), subtelocentric (st) and acrocentric (a).

### Reconstruction of ancestral characters using the Mesquite software

We performed a reconstruction of the ancestral chromosome number (2n) and NOR pattern using Mesquite software (Maddison and Maddison, 2011). For that, we incorporated the molecular species-level phylogeny of Doradidae and two outgroups from Auchenipteridae (its sister group), *Trachelyopterus galeatus* Linnaeus, 1766 and *Ageneiosus inermis* Linnaeus, 1766 (Arce et al., 2013). This study encompassed three datasets that included two mitochondrial DNA fragments (COI, n= 39 and 16S, n=41) and one nuclear DNA fragment (Rag 1, n=37) from previous studies available in online databases Genbank (Table 3). We reconstructed the phylogenetic relationships using Maximum Likelihood (ML) in the software packages RAxML-HPC v. 8.2.10 (Stamatakis, 2014) performed in the CIPRES Science Gateway 3.3 (http://www.phylo.org/index.php/portal/).

**Table 3 -:** Molecular (GenBank access numbers of genes used in the phylogenetic reconstruction) and cytogenetic data (diploid number and NOR pattern) used by the Mesquite software to estimate the ancestral diploid number and NORs pattern for Doradidae. Legend: Rag1= recombination activating gene 1; Co1= cytochrome c oxidase subunit 1; 16S= ribosomal RNA 16S; 2n= diploid number; NOR= nucleolar organizator region.

Species	Molecular data identifier			Cytogenetic data			
	Rag1	Co1	16s	Source	2n	NORs Pattern	Source
*Trachelyopterus galeatus*	-	EU490848.1	JX899742.1	Genbank	58	Simple NORs (Two sites)	Lui *et al*. (2010)
*Ageneiosus inermis*	KC555823.1	-	KC555843.1	Genbank	56	Simple NORs (Two sites)	Lui *et al*. (2013)
*Anadoras* sp. “araguaia”	KC555726.1	-	KC555850.1	Genbank	56	Simple NORs (Two sites)	Baumgärtner *et al*. (2018)
*Physopyxis ananas*	KC555793.1	KC555674.1	KC555928.1	Genbank	-	-	-
*Scorpiodoras heckelli*	KC555813.1	KC555695.1	KC555948.1	Genbank	-	-	-
*Hypodoras forficulatus*	KC555747.1	KC555619.1	KC555877.1	Genbank	-	-	-
*Astrodoras asterifrons*	KC555729.1	KC555597.1	KC555855.1	Genbank	-	-	-
*Amblydoras nheco*	KC555724.1	KC555642.1	KC555897.1	Genbank	-	-	-
*Acanthodoras* sp.2	KC555714.1	KC555580.1	KC555837.1	Genbank	-	-	-
*Wertheimeria maculata*	KC555822.1	KC555709.1	KC555963.1	Genbank	58	Simple NORs (Two sites)	Takagui *et al*. (2019)
*Kalyptodoras bahiensis*	KC555748.1	KC555622.1	KC555878.1	Genbank	58	Simple NORs (Two sites)	Takagui *et al*. (2019)
*Franciscodoras marmoratus*	KC555741.1	KC555610.1	KC555868.1	Genbank	58	Simple NORs (Two sites)	Takagui *et al*. (2019)
*Agamyxis pectinifrons*	KC555718.1	KC555584.1	KC555841.1	Genbank	-	-	-
*Rhyncodoras woodsi*	KC555810.1	KC555693.1	KC555946.1	Genbank	-	-	-
*Orinocodoras eigenmanni*	-	KC555664.1	KC555918.1	Genbank	-	-	-
*Rhinodoras dorbignyi*	KC555807.1	KC555690.1	KC555943.1	Genbank	58	Simple NORs (Two sites)	Baumgärtner *et al*. (2018)
*Pterodoras granulosus*	KC555802.1	KC555686.1	KC555939.1	Genbank	58	Simple NORs (Two sites)	This study
*Doraops zuloagai*	KC555736.1	KC555604.1	KC555862.1	Genbank	-	-	-
*Oxydoras niger*	KC555791.1	KC555672.1	KC555926.1	Genbank	58	Simple NORs (Two sites)	This study
*Centrochir crocodilli*	KC555731.1	KC555599.1	KC555861.1	Genbank	-	-	-
*Platydoras hancockii*	KC555798.1	KC555679.1	KC555933.1	Genbank	58	Multiple NORs (Four sites)	This study
*Platydoras costatus*	KC555797.1	KC555678.1	KC555932.1	Genbank	58	Simple NORs (Two sites)	Milhomem *et al*. (2008)
*Platydoras armatulus*	KC555795.1	KC555676.1	KC555930.1	Genbank	58	Simple NORs (Two sites)	Baumgärtner *et al*. (2018)
*Centrodoras brachiatus*	KC555733.1	KC555601.1	KC555858.1	Genbank	58	Simple NORs (Two sites)	This study
*Lithodoras dorsalis*	KC555763.1	KC555639.1	KC555895.1	Genbank	-	-	-
*Megalodoras goyanensis*	KC555764.1	KC555640.1	KC555896.1	Genbank	-	-	-
*Ossancora punctata*	KC555788.1	KC555670.1	KC555924.1	Genbank	66	Simple NORs (Two sites)	This study
*Doras higuchii*	KC555738.1	KC555606.1	KC555864.1	Genbank	-	-	-
*Trachydoras paraguayensis*	KC555818.1	KC555704.1	KC555958.1	Genbank	56	Simple NORs (Two sites)	Baumgärtner *et al*. (2016)
*Trachydoras steindachneri*	-	KC555708.1	KC555962.1	Genbank	60	Simple NORs (Two sites)	This study
*Anduzedoras oxyrhynchus*	KC555728.1	KC555594.1	KC555852.1	Genbank	-	-	-
*Leptodoras cataniae*	KC555750.1	KC555624.1	KC555882.1	Genbank	58	Simple NORs (Two sites)	Takagui *et al*. (2017b)
*Tenellus ternetzi*	KC555783.1	KC555661.1	KC555915.1	Genbank	58	Simple NORs (Two sites)	Milhomem *et al*. (2008)
*Tenellus leporhinus*	KC555773.1	KC555653.1	KC555907.1	Genbank	58	Simple NORs (Two sites)	Takagui *et al*. (2017b)
*Nemadoras elongatus*	KC555765.1	KC555643.1	KC555898.1	Genbank	-	-	-
*Tenellus trimaculatus*	KC555778.1	KC555656.1	KC555910.1	Genbank	58	Simple NORs (Two sites)	Takagui *et al*. (2017b)
*Hassar wilderi*	KC555744.1	KC555616.1	KC555874.1	Genbank	58	Simple NORs (Two sites)	Takagui *et al*. (2017b)
*Hassar orestis*	KC555743.1	KC555615.1	KC555873.1	Genbank	58	Simple NORs (Two sites)	Milhomem *et al*. (2008)
*Ossancora fimbriata*	-	KC555667.1	KC555921.1	Genbank	-	-	-
*Opsodoras morei*	KC555781.1	KC555659.1	KC555913.1	Genbank	-	-	-
*Hemidoras stenopeltis*	KC555746.1	KC555618.1	KC555876.1	Genbank	58	Simple NORs (Two sites)	This study

The ancestral state was inferred using Maximum Likelihood analysis and Markov model 1 state (Mk1), which considers that all changes are equally possible. The cytogenetic data used in the reconstruction were obtained from the literature (Table 3), including the data of the present study. The characters were treated as non-ordered and multi-state, with five states being considered for the diploid number (data absent; 2n=56; 2n=58; 2n=60; 2n=66) and three states for the NORs pattern (data absent; Simple NORs, Multiple NORs). The likely ancestor character was determined for each node, and the probabilistic values were organized in Table 4.

**Table 4 -:** Probabilistic values calculated after, maximum likelihood ancestral state reconstructions of diploid number and NORs pattern, based on Mk1 model using the Mesquite software in Doradidae species. The values highlighted in red, are the most probably ancestral character for each node.

Nodes	Diploid Numbers					NORs Pattern			Clades
	Undefined	2n=56	2n=58	2n=60	2n=66	Undefined	Simple	Multiple	
1	35.4	32.7	22.3	4.7	4.7	23.3	69.5	7.0	Doradoidea
2	11.7	45.4	36.8	2.9	2.9	3.4	94.9	1.6	Auchenipteridae
3	64.7	22.2	8.9	2.1	2.1	43.5	51.7	4.6	Doradidae
4	63.1	29.8	3.6	1.6	1.6	43.0	53.2	3.7	Astrodoradinae
5	95.8	2.6	0.6	0.4	0.4	89.5	8.4	2.0	
6	99.2	0.3	0.1	0.1	0.1	97.3	1.7	0.8	
7	99.2	0.3	0.1	0.1	0.1	98.7	0.6	0.5	
8	99.2	0.3	0.1	0.1	0.1	99.0	0.6	0.4	
9	85.1	4.7	7.8	1.0	1.0	65.9	30.6	3.4	
10	75.6	2.3	19.5	1.2	1.2	55.6	40.7	3.6	
11	6.4	0.7	91.3	0.6	0.6	0.8	89.4	1.9	Wertheimerinae
12	0.5	0.1	98.9	0.1	0.1	0.1	97.7	0.07	
13	90.1	0.6	8.1	0.5	0.5	78.4	18.9	2.6	
14	86.2	0.6	11.9	0.6	0.6	73.0	23.9	0.2	Doradinae
15	92.2	0.4	6.4	0.4	0.4	84.2	13.5	2.1	
16	84.1	0.8	13.3	0.8	0.8	74.0	23.2	2.6	
17	59.4	0.9	37.7	0.9	0.9	46.4	50.1	3.4	
18	57.4	0.9	39.5	0.9	0.9	47.1	49.7	3.1	
19	33.4	0.8	63.8	0.8	0.9	2.0	77.0	2.7	
20	38.7	0.9	58.2	0.9	1.1	23.3	73.3	3.2	
21	41.1	1.0	55.5	1.0	1.1	34.9	60.2	4.8	
22	3.5	0.4	95.0	0.4	0.4	9.5	83.2	0.7	
23	0.3	0.1	99.2	0.1	0.1	5.0	74.8	20.1	
24	41.7	1.6	52.4	1.6	2.5	20.0	77.8	2.8	
25	42.6	1.3	53.1	1.3	1.5	26.2	70.6	3.0	
26	94.2	0.5	4.1	0.5	0.5	88.6	9.4	1.9	
27	41.9	4.4	40.7	4.4	8.5	15.2	82.3	2.4	
28	54.1	2.9	13.4	2.9	26.5	24.4	72.7	2.7	
29	26.9	10.6	46.7	10.6	4.9	0.6	91.4	1.7	
30	10.0	35.2	15.7	35.2	3.7	1.2	98.0	0.06	
31	22.4	2.9	69.8	2.9	1.8	9.9	87.9	2.0	
32	28.1	1.6	67.1	1.6	1.3	20.6	76.6	2.6	
33	13.5	0.9	83.6	0.9	0.7	8.9	89.0	2.0	
34	15.7	0.8	81.6	0.8	0.8	14.6	82.7	2.6	
35	0.1	0.2	98.0	0.2	0.2	2.2	96.7	0.9	
36	57.0	1.0	39.8	1.0	1.0	56.0	40.7	3.2	
37	55.5	1.0	41.3	1.0	1.0	53.9	43.0	3.0	
38	7.7	0.5	90.6	0.5	0.4	7.2	90.9	1.7	
39	14.5	0.8	82.9	0.8	0.8	18.6	78.7	2.5	
40	0.6	0.1	98.8	0.1	0.1	1.3	97.9	0.7	

## Results

**Figure 1 -:**
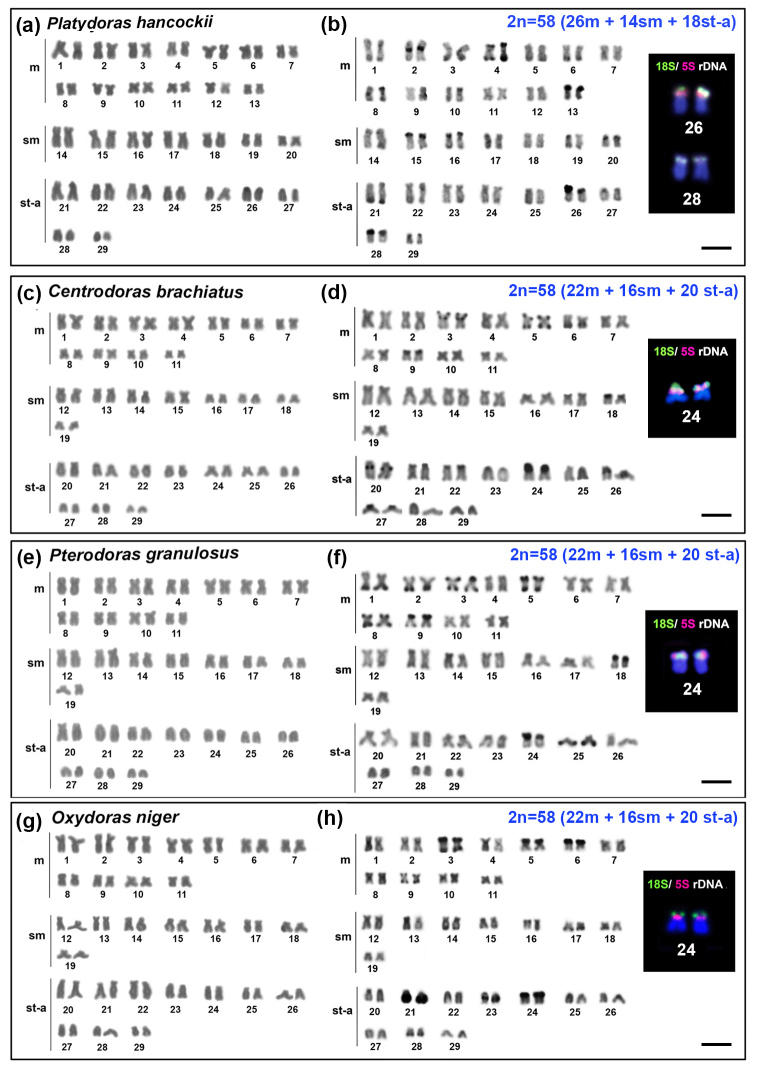
Karyotypes of the Doradinae subfamily: *Platydoras hancockii* (a) Giemsa, (b) C-band; *Centrodoras brachiatus* (c) Giemsa, (d) C-band; *Pterodoras granulosus* (e) Giemsa, (f) C-band; *Oxydoras niger* (g) Giemsa, (h) C-band. The boxes contain the chromosome pairs bearing the 18S and 5S rDNA rDNA sites. The scale bar corresponds at 10 µm.

***Platydoras hancockii**:* Valenciennes 1840: had 2n=58 chromosomes (26m + 14sm + 18st-a) (Figure 1A). Heterochromatin was detected on short arm of the pairs 13, 15, 16, 20, 26, 28 and on long arm of the pair 3, 6 and 21; on both arms of the pairs 4 and 8; and in interstitial position (near to the centromere) on short arms of the pair 2 (Figure 1B). The FISH using the 18S rDNA probes, evidenced multiple sites in terminal position on short arms of the pairs 26 and 28. The FISH with 5S rDNA probes, revealed hybridized sites on the short arm of the pair 26, the same chromosome pair were the 18S rDNAs sites were detected (Figure 1 box).

***Centrodoras brachiatus***: had 2n=58 chromosomes (22m + 16sm + 20st-a) (Figure 1C). C-banding evidenced heterochromatin blocks on short arms of the pairs 9, 18, 22, 24 and 27; on long arms of the pair 6; interstitial blocks on long arms of the pairs 20 and 26; in both arms of the pair 5; in pericentromeric and terminal regions on short arm of the pair 3 (Figure 1D). The FISH with rDNA probes, evidenced the presence of 18S rDNA sites and 5S rDNA sites on short arm of the pair 24, being that the 18S rDNA sites are located on terminal position, whereas 5S rDNA sites occurs in interstitial position, near to the centromere (Figure 1 box).

***Pterodoras granulosus***: had 2n=58 chromosomes (22m + 16sm + 20st-a) (Figure 1E). Heterochromatic blocks were detected on short arms of the pairs 9, 18, 24; long arms of the pairs 1, 2, 25 and on both arms of the pairs 3, 5, 8 (Figure 1F). The FISH with rDNA probes revealed the presence of DNA 18S rDNA in terminal position on short arm of the pair 24, adjacent to the 5S rDNA sites (Figure 1 box).

***Oxydoras niger**:* had 2n=58 chromosomes (22m + 16m + 20st-a) (Figure 1G). C-banding evidenced heterochromatic blocks on short arms of the pairs 5, 6, 24 and on long arm of the pairs 14, 17, 21, 28 on both arms of the pairs 3, 9 and in interstitial position on long arms of the pair 23 (Figure 1H). FISH also revealed 18S and 5S rDNA sites on short arms of the pair 24, being the NORs sites in terminal position, while 5S rDNA sites was detected interstitially, near to the centromere (Figure 1 box).

**Figure 2 -:**
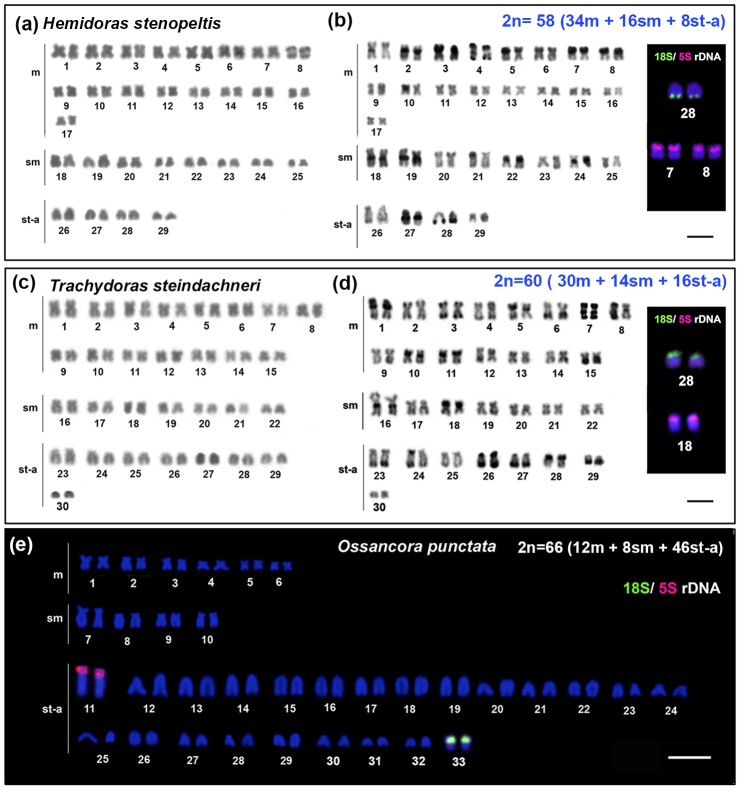
Karyotypes of the Doradinae subfamily “fimbriate barbells thorny catfishes”: *Hemidoras stenopeltis* (a) Giemsa, (b) C-band; *Trachydoras steindachneri* (c) Giemsa, (d); The boxes contain the chromosome pairs bearing the 18S and 5S rDNA rDNA sites. (e) karyotype of *Ossancora punctata* after FISH with 18S and 5S rDNA probes. The scale bar corresponds at 10 µm.

***Hemidoras stenopeltis*** Steindachner 1881: had 2n=58 chromosomes (34m + 16sm + 8a) (Figure 2A). The heterochromatin was detected in terminal position on short arms of the pairs 3, 5, 7, 8, 21, 22; on long arms of the pair 28; in pericentromeric region of the pairs 2, 18, 19, 24, 27 and on both arms of the pair 4 (Figure 2B). The 18S rDNAs sites showed hybridized signals in terminal position on long arms of the pair 28, whereas 5S rDNA sites were detected on short arms of the pairs 7 and 8 (Figure 2 boxed).

***Trachydoras steindachneri*** Perugia 1897: had 2n=60 chromosomes (30m + 14sm + 16a) (Figure 2C). C-banding evidenced terminal heterochromatic blocks on short arms of the pairs 10, 11, 18 and 28; on long arms of the pairs 6, 15, 23, 24, 27, 29; in pericentromeric regions in the pairs 5, 10, 11 and 16; on both arms of the pairs 3, 7, 26; in pericentromeric and terminal position on short arm of the pair 1 (Figure 2D). FISH revealed 18S rDNA sites on short arms of the pair 28 and 5S rDNA sites on short arms of the pair 18 (Figure 2 boxed).

***Ossancora punctata***: had the karyotype and heterochromatin pattern previously described by Takagui *et al*. (2017a) and shows 2n=66 chromosomes, the largest diploid number in the family. Here, we present unpublished data about the distribution of rDNA sites in the karyotype of this species. The rDNA sites were detected in distinct chromosomal pairs, but both located in terminal position and on short arms, being that the 18S rDNA sites in the pair 33 and 5S rDNA sites in the pair 11 (Figure 2E).

## Reconstruction of ancestral chromosome characters in Doradidae clades

### (a) Diploid number

**Figure 3 -:**
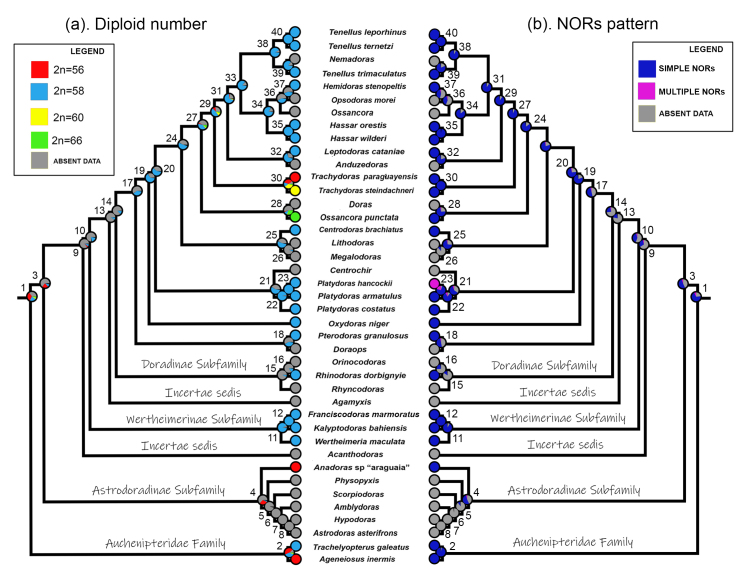
Mirror trees showing maximum likelihood ancestral state reconstructions of diploid number and NORs pattern, based on Mk1 model using the Mesquite software. This evolutionary analysis integrated cytogenetic data available for Doradidae species (including the present study) and two Auchenipteridae species (sister group) with sequences of two mitochondrial DNA fragments (COI and 16S) and one nuclear DNA fragment (Rag 1) obtainad from the molecular phylogeny of Arce *et al*. (2013).

When we integrated the diploid number data available for thorny catfishes with the molecular phylogenetic analysis carried out by Arce et al. (2013), we observed that the probabilistic values obtained for the basal nodes are low and very close to each other. Thus, it is not yet possible to determine which would be the ancestor state for diploid number for the Doradidae family. Our data indicate that both 2n=56 and 58 chromosomes might be considered equally parsimonious ancestral conditions for Doradoidea (node 1), Auchenipteridae (node 2) and Doradidae (node 3). Moreover, stablishing the ancestral 2n in Astrodoradinae was hampered by the low number of species cytogenetically analyzed so far. On the contrary, the 2n=58 chromosomes in Wertheimerinae is the ancestral condition with 99.9% of support. The lack of chromosomal data in basal clades of Doradinae also made it impossible to define which 2n would be the ancestral condition for the subfamily (node 14), as well as for other terminal clades (nodes 15, 16, 17, 18, 26, 28, 36, 37). *Doras* + *Ossancora* and *Trachydoras* clades have a greater 2n variability reported in its analyzed species; hence, increasing the studies in other species of these genera is required prior to reconstructing their likely ancestral 2n with accuracy (Figure 3, Table 4). 

### (b) NORs pattern

Our analyses show that simple NORs pattern is likely to be the ancestral condition for Doradidae, however, the value that supports such condition (51,7%) is not significantly high and sufficient to confirm this hypothesis. Only one species from Astrodoradinae has cytogenetic data available; therefore, insufficient samples to define the pattern of NORs for this subfamily (nodes 4,5,6,7,8). On the other hand, simple NORs was confirmed as an ancestral condition with high support values (89,4% and 97,7%) in Wertheimerinae. Simple NORs was defined as an ancestral trait in most clades of Doradinae, except for the basal clades (nodes 14,15 e 16) and a part of the apical ones (26, 36 e 37) (Figure 3, Table 4).

## Discussion

The origin of the current karyotype diversity in Doradidae has been assigned to numerical (Robertsonian translocations), structural (pericentric inversions) and different mechanisms of repetitive DNA dispersion (Baumgärtner et al., 2018; Takagui et al., 2019). The hypothesis that the contemporary thorny catfishes diversified from an ancestor with a karyotype composed by 58 chromosomes and simple NORs has been inferred by several studies (Eler et al., 2007; Milhomem et al., 2008; Baumgärtner *et al*., 2016; Takagui *et al*., 2017a, 2017b; Baumgärtner *et al*., 2018; Takagui *et al*., 2019). In fact, these characteristics occur in most Doradidae species, as well as in related groups, such as Auchenipteridae (Lui et al., 2010, 2013a, 2013b, 2015; Felicetti et al., 2021). In this scenario, a very intriguing question emerge: would the prevalence of 2n=58 chromosomes and simple NORs in Doradidae and Achenipteridae (sister group) be enough arguments to support such characteristics as plesiomorphies in the family? 

The reconstruction analysis of ancestral characters based on the likelihood method and Markov MK1 model imply that none of the evaluated characteristics (diploid number and NORs) had sufficient support values to be confirmed as plesiomorphic conditions for Doradidae. In fact, the hypothesis of 2n=58 chromosomes and simple NORs as ancestral states is applicable solely to Wertheimerinae and part of Doradinae clades, groups in which most of the cytogenetic studies are concentrated. Therefore, this would be the reason that led some authors to attempt to define ancestral conditions for the whole family. The uncertainty of the ancestral patterns for Doradidae is a reflect of the paucity of karyotype data in the basal-most clades. Cytogenetic studies in Astrodoradinae, as well as in *Acanthodoras* and *Agamyxis* will be required to confirm or refute the ancestral karyotype hypothesis previously claimed for the group.

The presence or absence of fimbriate barbells, divides Doradinae into two large clades (Birindelli, 2014), also supported by molecular data (Arce et al., 2013). From a cytogenetic perspective, the ancestral karyotype remained highly conserved among the non-fimbriate barbells thorny catfishes, such as *Platydoras*, *Rhinodoras*, *Pterodoras*, *Oxydoras* and *Centrodoras*, where all the species have 2n=58, however, most of them has variable chromosomal morphology (Table 1). These differences have been mainly attributed to pericentric inversions, which are considered, in a general context, the most important rearrangement for karyotypic diversification in Doradidae (Eler et al., 2007, Milhomem et al., 2008; Baumgärtner et al., 2018; Takagui et al., 2019). From an evolutionary point of view, the pericentric inversions promote genetic variability and could be involved with reproductive isolation, and therefore, contribute to the speciation process (King, 1993; Noor et al., 2001), as already reported in several fish groups such as *Loricariichthys* (Takagui *et al*., 2014), *Apteronotus* (Takagui *et al*., 2017c; Fernandes et al., 2017), *Chrenicichla* (Frade et al., 2019), *Boulengerella* (de Souza et al., 2017), *Brachyhypopomus* (Cardoso *et al*., 2018), *Exallodontus* and *Propimelodus* (Terra et al., 2019).

The large thorny catfishes *Centrodoras brachiatus*, *Pterodoras granulosus* and *Oxydoras niger*, shared the same diploid number, karyotypic formulae and rDNAs sites array. These similarities in their karyotypes reinforce the close relationship among these species, which are cytogenetically distinguished only by the distribution of the heterochromatin. According to Motta-Neto *et al*. (2019), the karyotype stasis (in different levels), is a multifactorial process resultant by phylogenetic (recent or ancient radiation), biological (dispersion capacity, populational size, habitat preferences), and biogeographic contexts (presence of geographic barriers, stable environments). The three thorny catfishes species aforementioned, constitute demes with a high number of individuals that seasonally perform migration movements during the reproductive period (Goulding, 1980; Agostinho *et al*., 2003; Birindelli and Sousa 2017). Thus, we can infer that the population size, high vagility, phylogenetic proximity and stabilizing natural selection mechanisms, may be decisive factors that act synergistically, underscoring the chromosome conservatism in this group. This correlation, also occurs in other Neotropical fish species, such as Anostomidae (Martins and Galetti, 1998), Prochilodontidae (Voltolin et al., 2013), Tetraodontidae (Viana et al., 2017) and in large catfishes of the subfamily Sorubiminae (Swarça et al., 2013).

A greater cytogenetic variability was observed among the fimbriate-barbells clade when compared to the other clades placed into Doradinae subfamily (Table 1). This group shows different diploid numbers ranging from 2n=56 to 2n=66, supernumerary chromosomes (Takagui et al., 2017a) and a unique ZZ/ZW differentiated sex chromosome system (Takagui *et al*., 2017b). Derived diploid numbers was observed in *Trachydoras paraguayensis*, which has 2n=56 chromosomes, originated from a chromosomal fusion (Baumgärtner et al., 2016), *Trachydoras steindachneri* with 2n=60 product of one centric fission (present study) and *Ossancora punctata* with 2n=66 chromosomes, which possibly arose due to four centric fissions and multiple pericentric inversions from an ancestral karyotype composed by 58 chromosomes (Takagui *et al*., 2017b). Such diversity may be interpreted as a reflect of the non-migratory behavior. These species occur mainly in sandbanks, at the deep of the main channels of large rivers or in marginal lagoons, associated with floating or riparian vegetation (Birindelli and Sousa, 2017). The sedentarism and microhabitat preference associated with small population sizes, are characteristics that may be enhancing the chromosomal rearrangements fixation along the same hydrographic basin. This hypothesis has been corroborated by different groups of fish widely distributed in the Amazon basin, as seen in *Ancistrus* (de Oliveira et al., 2009), *Farlowella* (Marajó et al., 2018) and in the species complex *Bunocephalus coracoideous* (Ferreira et al., 2017).

Simple NORs in terminal position, appears as a plesiomorphic condition with high support values in Doradinae, although it remains an issue to be further investigated in most clades of the subfamily. In the *Platydoras* clade, a multiple NORs system was observed only in *Platydoras hancockii,* such configuration apparently represents a derived condition in Doradidae and hitherto particular to this species. The spreading of NORs sites between different chromosomes has often been related to the presence of transposable/mobile elements, which may insert itself in regions of DNAr 18S and spread them to other chromosomal sites (Raskina et al., 2004; Eickbush and Eickbush, 2007; Porto et al., 2014, among others). Another plausible and widely discussed possibility is the occurrence of non-reciprocal translocations involving terminal or sub-terminal segments (Hirai, 2020; Takagui *et al*. 2020;). In this case, the proximity of these regions during the meiotic interphase (Rabl’s Model), would facilitate the exchange of 18S DNAr segments in the terminal regions between non-homologous chromosomes (Cremer et al., 1982; Schweizer and Loidl 1987). 

The localization of 18S and 5S rDNA sites in the same chromosome pair is unusual in closely related groups to Doradidae family: few Aspredinidae species possess such condition (Ferreira et al., 2017, 2020), also, the sister family Auchenipteridae has no evidence of syntenic rDNA sites (Lui et al., 2010, 2013a, 2013b, 2015; Felicetti et al., 2021). According to Baumgärtner et al., 2018), the presence of 18S and 5S rDNA sequences adjacently organized on short arms of one subtelocentric pair could indeed represent an ancestral condition for Doradidae species. Recently, Takagui et al. (2019) also revealed a sole subtelocentric pair bearing 18S and 5S rDNA for all Wertheimerinae species, reinforcing this trait as a plesiomorphic condition, once Wertheimerinae is considered one of the most ancient lineages among thorny catfishes, sister group to Doradinae. Our data also highlights that this association is maintained for at least the large thorny catfishes species in Doradinae, as seen in *P. granulosus*, *P. hancockii*, *O. niger* and *C. brachiatus*. However, syntenic breakage events might have occurred at the very beginning of fimbriate-barbell thorny catfishes differentiation. Notably, excepting *Ossancora eigenmanni,* all species of this clade do not have 18S and 5S rDNA sharing the same location on a chromosome pair.

The 5S rDNA distribution, when compared to 18S rDNA, is so much more variable and unstable, holding numerical and structural variability and also representing an excellent cytotaxonomic marker for Doradidae species (Table 1). For instance, *Platydoras hancockii* and *Platydoras armatulus* (Baumgärtner et al., 2018) can be easily differentiated from each other by the presence of differential 5S rDNA sites, and the same occurs among *Tenellus* species (Takagui et al., 2017b) and in Wertheimerinae (Takagui *et al*., 2019). In Auchenipteridae, 5S rDNA sites distribution pattern has also been useful to characterize species of *Tatia* (Lui et al., 2013a), as well as populations of *Trachelyopterus galeatus* (Lui *et al*., 2009; Lui *et al*., 2010). In general, most variability in the 5S rDNA distribution is attributed to the presence of different repetitive DNA classes in non-transcribed regions (NTS) of 5S rDNA, which is common in fish groups, including transposable elements such as LINES, SINES and non-LTR retrotransposons (Rebordinos et al., 2013; Gouveia et al., 2017), histones DNA (Hashimoto et al., 2011; Piscor et al., 2018), small nuclear RNA (Silva et al., 2015) as well as different microsatellites motifs (Gouveia *et al*., 2017). 

Our results combined, shed light on the karyotype diversification of Doradinae, the most representative subfamily among thorny catfishes. Our cytogenetic analyses and reconstruction of ancestral states brought important new insights into evolutionary pathways traced by doradids, providing thus, two striking evolutionary trajectories: low variation and conservatism of several chromosomal features in large thorny catfishes (non-fimbriate barbells) and remarkable diversity in tiny species from fimbriate barbells group, often mediated by dynamic behaviors and complex evolutionary processes, still far from being fully solved. However, the available data suggest that the main mechanisms responsible for the current karyotype variability are: pericentric inversions (Baumgärtner et al., 2018), chromosomal fusions (Baumgärtner *et al*., 2016), centric fissions (Takagui et al., 2017a), paracentric inversion (Takagui *et al*., 2017b) and differential dispersion of heterochromatin regions driven by transposable elements activity (Takagui *et al*., 2019). 
